# Editorial: Heart Valve Tissue Engineering: Are We Ready for Clinical Translation?

**DOI:** 10.3389/fcvm.2021.658719

**Published:** 2021-05-13

**Authors:** Jesper Hjortnaes, M. M. Mokhles, J. J. M. Takkenberg, C. V. C. Bouten

**Affiliations:** ^1^Department of Cardiothoracic Surgery, Leiden University Medical Center, Leiden, Netherlands; ^2^Department of Cardiothoracic Surgery, University Medical Center Utrecht, Utrecht, Netherlands; ^3^Department of Cardiothoracic Surgery, Erasmus Medical Center, Rotterdam, Netherlands; ^4^Department of Biomedical Engineering and Institute for Complex Molecular Systems, Eindhoven University of Technology, Eindhoven, Netherlands

**Keywords:** tissue engineering, valve, biomedical engineering, valve disease, biotechnology

It has been 25 years since the first seminal paper on heart valve tissue engineering was first published by Dr. Shinoka and Dr. Breuer. Lead by Dr. Mayer, and in collaboration with Dr. Vacanti and Dr. Langer—all of whom are now considered visionary pioneers in their field— they collectively worked together to publish the first results of a tissue engineered heart valve leaflet in a lamb model (1). A new research field quickly developed, culminating into an active, ambitious, and driven research community across the globe motivated to develop a “living” prosthesis that outperforms inert valvular implants. It is only fitting to reflect on more than two decades of research in the pursuit of developing the perfect tissue engineered valve, particularly when put into the context of an immense growth in scientific output and topical journals in the “heart valve tissue engineering” realm (2). The original purpose of this endeavor is to develop a prosthetic valve that is tailor-made, autologous, and which is able to exhibit adaptation to and grow with a patient. By employing these specific characteristics, a tissue engineered heart valve would overcome the well-established complications associated with current valve prosthesis, such as limited durability with bovine or porcine heart valve prostheses or bleeding-disorders and thromboembolic complications attributed to mechanical heart valves. Moreover, as a living growing valve, a tissue-engineered heart valve could provide a solution for patients with congenital heart disease who will need repeated heart valve replacement over the course of their lives.

Albeit relentless efforts of finally being able to safely and reliably implant tissue engineered heart valves into patients, the honest evaluation is that we are not ready yet. In light of this, important questions arise: Where do we stand today? To what extent have we met the expectations of the scientific community? Have we let our patients down? And what remains to be done? What are the prospects for translation into clinical practice? And if so, when? And with whom? Where does our task as scientists stop and where will responsibility of others (regulatory bodies, companies, etc.) start?

We believe our common purpose can be fulfilled. This Research Topic has collected contributions by some of the pioneers of heart valve tissue engineering and reflects on the hurdles we as a community still need to overcome in the transition to successfully translate tissue engineered heart valves to patients.

An argument put forward by Zilla et al. in this Research Topic is that research needs to focus on feasibility outcomes tailored to the patients we are trying to help. They argue elegantly that the association between clinical purpose, human pathobiology, and laboratory-based solutions may be waning and urge a return to patient-centered development of a tissue engineered valve. A breakthrough in biomaterial driven healing of tissue could be achieved by a concerted effort to understand the underlying principles of prosthetic healing in patients Zilla et al..

Following this line of reasoning, from a surgical point of view, the tissue engineered constructs we aim to develop need to be hemostatic, hold sutures, and be resistant to pressure and stress, while also being tissue-friendly and resistant to thrombosis. However, as Durko et al. rightly address, and contrary to the common evaluation of a surgical product in a patient, a tissue engineered heart valve is not a “final product,” as their properties and behavior change upon implantation. This makes the interpretation of *in-vivo* outcomes of tissue engineered heart valves quite challenging in its own right Durko et al..

An important theme, which has harnessed a great deal of focus, in particular with regards to studies that have implanted tissue engineered heart valves in large animal models, is the pre-clinical assessment of these substitutes. In short, the challenge can be defined into a singular question: When are tissue engineered valves deemed good enough for clinical use? Zhang et al. impressively delineate not only the developmental hurdles of such quality control, but also show the challenges regulatory approval of tissue engineered heart valves may face. The authors propose a much-welcomed road map that could lead to clinical translation in patients Zhang et al..

In light of the previous arguments put forward, if we are to understand and direct prosthetic healing in patients, the natural healing and homeostasis of tissue needs to be better understood. In particular, to what extent do we understand valvular biology enough so we can replicate it and control it to fabricate a tissue engineered heart valve that durably maintains valve function? To that end, Grande-Allen and Chester provide an excellent overview where such knowledge stands in relation to valve tissue engineering. Their work provides a state-of-the-art perspective on the biological functions of heart valve cells, the interaction between cells and their environment, and how these concepts relate to tissue engineering of heart valves *in-vitro, in-vivo*, and *in-situ* Chester and Grande-Allen.

In conclusion, it is clear that challenges remain to be addressed in translating heart valve tissue engineering to our patients. However, the field does seem to have entered the beginning of the end, and translation may be on the horizon. As put forward in this Research Topic, these challenges revolve around (a) achieving a better understanding of valve tissue healing and valve biology, (b) harnessing that knowledge into reproducible biomaterial-driven healing and regeneration, and (c) eventually developing a sound and safe regulatory control of the technology. The latter also undoubtedly needs to be evaluated in terms of cost-effectiveness (3). A catalyzing effort could be mounted by bringing our community closer together. We could develop a renewed sense of purpose by avoiding competition and supporting collaboration across the globe, preventing repetitious mistakes and re-inventing the wheel. To such an end, collective databases may be developed to evaluate results of *in-vitro* and animal studies more effectively and collectively, as is becoming common in neighboring fields. This could lead to well-designed randomized large animal studies with gold standard imaging techniques in our community. Effective organization of such a global community is vital to achieving success. The paradox of science seems to apply to our community as well; “the more we know, the more questions we have.” We can only underline the advice put forward by all contributing authors to this Research Topic: Let us not lose sight of what is needed for focused clinical translation: the patient!

## Author Contributions

All authors listed have made a substantial, direct and intellectual contribution to the work, and approved it for publication.

## Conflict of Interest

The authors declare that the research was conducted in the absence of any commercial or financial relationships that could be construed as a potential conflict of interest.
